# The role of neoadjuvant chemotherapy before radical surgery in stage IB2/IIA2 squamous cell cervical cancers

**DOI:** 10.1186/s12905-024-03215-8

**Published:** 2024-06-22

**Authors:** Aysun ALCI, Okan Aytekin, Burak Ersak, Fatih Kilic, Okan Oktar, Cakır Caner, Vakkas Korkmaz, Gunsu Kimyon Comert, İlker Selcuk, Tayfun Toptas, Nurettin Boran, Tolga Tasci, Alper Karalok, Derman Basaran, Ozlem Moraloglu Tekin, Yaprak Engin Ustun, Taner Turan, Isin Ureyen

**Affiliations:** 1 Department of Gynecological Oncology, Antalya Health Science University Training and Research Hospital, Varlik m., Kazım Karabekir street, Antalya, 07100 Turkey; 2 Department of Gynecological Oncology, Ankara Health Science University Bilkent City Hospital, Ankara, 0600 Turkey; 3 Department of Gynecological Oncology, Ankara Health Science University Etlik Zubeyde Hanim Women’s Health and Research Hospital, Ankara, 0600 Turkey; 4 Department of Gynecological Oncology, Bahcesehir University Medical School Hospital, Ankara, 0600 Turkey; 5 Department of Gynecological Oncology, Losante Hospital, Ankara, 0600 Turkey; 6 Department of Gynecological Oncology, Hacettepe University Faculty of Medicine, Ankara, 0600 Turkey

**Keywords:** Neoadjuvant chemotherapy, Radical surgery, Cervical cancer

## Abstract

**Background:**

This study aimed to evaluate the outcomes of patients diagnosed with stage IB2/IIA2 cervical squamous cell carcinoma who underwent neoadjuvant chemotherapy (NACT) prior to radical hysterectomy compared to those who did not receive NACT before surgery.

**Materials and methods:**

This is a multicenter study including data of 6 gynecological oncology departments. The study is approved from one of the institution’s local ethics committee. Patients were stratified into two cohorts based on the receipt of NACT preceding their surgical intervention. Clinico-pathological factors and progression-free survival were analyzed.

**Results:**

Totally 87 patients were included. Lymphovascular space invasion (LVSI) was observed as 40% in the group receiving NACT, while it was 66.1% in the group not receiving NACT (*p* = 0.036). Deep stromal invasion (> 50%) was 56% in the group receiving NACT and 84.8% in the group not receiving NACT (*p* = 0.001). In the univariate analysis, application of NACT is statistically significant among the factors that would be associated with disease-free survival. Consequently, a multivariate analysis was conducted for progression-free survival, incorporating factors such as the depth of stromal invasion, the presence of LVSI, and the administration of NACT. Of these, only the administration of NACT emerged as an independent predictor associated with decreased progression-free survival. (RR:5.88; 95% CI: 1.63–21.25; *p* = 0.07).

**Conclusions:**

NACT shouldn’t be used routinely in patients with stage IB2/IIA2 cervical cancer before radical surgery. Presented as oral presentation at National Congress of Gynaecological Oncology & National Congress of Cervical Pathologies and Colposcopy (2022/ TURKEY).

## Introduction

Cervical cancer is the fourth most prevalent malignant tumour that affects women globally [1], with Squamous cell carcinoma being the most common (90%) histological subgroup of cervical cancer [2]. Adenocarcinoma and adenosquamous carcinoma are additional common histological variants of cervical cancer; however, reports of small cell carcinoma, sarcoma, lymphoma, and metastatic cancer are less common [3].

Global cancer statistics indicate that there were 604.000 newly diagnosed cases and 342.000 deaths attributed to cervical cancer [4]. The most relevant risk factor for cervical cancer is persistent human papillomavirus (HPV) infection, which is present in 98% of cases [5, 6]. Other risk factors include smoking, chronic immunosuppression, use of oral contraceptives, parity, early sexual activity, having multiple sexual partners, and a history of sexually transmitted infections [7, 8].

According to the 2014 staging criteria of FIGO (The International Federation of Gynecology and Obstetrics), carcinomas that are greater than 4 cm but are limited to the cervix are categorised as stage IB2. Stage IIA2 is defined by the presence of a tumour that exceeds 4 cm in size (clinically visible) and has the ability to invade the uterine serosa but does not extend to the pelvic side wall [9]. Survival rate in early stage (Stage IA1-IB1) non-bulky cervical cancer is as high as 80–90% [10–12], while the recurrence rate for stage IB2/IIA2 cervical cancer is 34% and the 5-year overall survival rate is reported as 70% [13].

Local-regional control is essential for long-term survival in cervical cancer patients and it is essential to identify the factors that modify the site of recurrence. Surgery and/or chemoradiotherapy are the standard approaches in the treatment of cervical cancer at the time of presentation and in accordance with the stage of the disease [14, 15]. While radical surgery is the primary approach for early-stage cervical cancer, chemoradiotherapy is recommended for locally advanced disease (FIGO 2014 stage IIB-IVA). Nevertheless, optimal treatment modality for women with stages IB2-IIA2 disease is controversial [16].

The oncogenesis of HPV primarily relies on the production of E6 and E7 proteins. HPV E6 and E7 oncoproteins induce carcinogenesis by suppressing the function of the retinoblastoma and p53 tumour suppressor genes [17]. A rational approach to treating cervical cancer is to reinstate reduced p53 levels by destroying the HPV E6 oncoprotein. An alternative approach involves the prevention of negative feedback inhibition of the MDM2 oncoprotein on p53 through the up-regulation of miR-605. It is reported that specifically, platinum-based NACT (neoadjuvant chemotherapy) strongly upregulates miR-605 [18].

Many studies demonstrated that NACT reduces micrometastases, shrinks tumor size and facilitates surgical resection in cervical cancer [19–22]. In this regard, administering NACT before radical surgery aims to reduce tumour size, enhance surgical resectability, minimise the risk factors of recurrence, and eventually improve survival rates [23]. Radiotherapy is not preferred when treating recurrences in people who have already undergone initial radiotherapy. Exenteration, a radical surgical procedure, is the primary approach for managing recurrence in these patients. Another objective of NACT is to reduce the necessity for adjuvant radiotherapy, therefore preserving radiotherapy as a viable therapeutic choice for pelvic recurrences while reassuring for a less invasive surgical approach [24].

The literature does not provide a definitive set of criteria for selecting patients who should receive neoadjuvant chemotherapy (NACT) for cervical cancer. Some authors advocate the use of NACT within the time frame of the study, while others state that the use of NACT is controversial [25, 26]. Consequently, each institution has developed its own procedures based on the existing literature. We conducted a retrospective analysis of the data from this group of patients in order to provide insight into this controversial issue.

The objective of this study was to assess the survival rates and identify the factors that influence the survival of patients diagnosed with stage IB2/IIA2 squamous cell cervical cancer who underwent either radical surgery alone or radical surgery following neoadjuvant chemotherapy (NACT). We anticipate that our findings will assist in the development of treatment strategies and follow-up approaches for patients with cervical cancer.

## Methods

The current retrospective, multicentre, observational study was conducted and authorised by the Ethics Committee of Ankara Health Science University Etlik Zubeyde Hanim Women’s Health and Research Hospital, Ankara, Turkey, according to the Declaration of Helsinki. (approval no: 08/19, dated June 05, 2020). The study was conducted by examining the hospital records and outpatient follow-up cards of patients who underwent surgery for stage IB2/IIA2 cervical cancer at six gynaecological oncology departments.

Patients who had stage IB2-IIA2, squamous histology, and did not have any preoperative imaging were excluded from the study. In the treatment of stage IB2- IIA2 cervical cancer, radical surgery, radical surgery after neoadjuvant chemotherapy may be preferred depending on the clinician’s approach. All patients were diagnosed with squamous cell cervical carcinoma through histological investigation.

The sensitivity and specificity of imaging techniques for detecting lymph node metastases vary in the literature. The computed tomography (CT) scan showed a sensitivity rate of 50% and a specificity rate of 92%, while the MRI scan exhibited a sensitivity rate of 56% and a specificity rate of 91%. On the other hand, the positron emission tomography-computed tomography (PET-CT) scan exhibited relatively higher sensitivity and specificity rates, reaching 82% and 95%, respectively [3]. Our study utilised ultrasound, CT, MRI, PET-CT, and their combinations in accordance with the facilities and protocols of the centre, following the imaging modalities indicated by the FIGO cervical cancer staging system [26, 27].

Patient consent was not obtained due to the retrospective nature of the study. However, treatment options were provided to patients in all six centres immediately upon diagnosis. Patients were provided with comprehensive information regarding these options, and all options were clarified in accordance with the most recent literature. Patient autonomy was not disregarded in this patient group, whose management is still under debate.

The study compared various clinical and pathological factors, including age, tumour size, quantity of metastatic lymph nodes, stage of cancer, infiltration of the parametrium, status of surgical margins, involvement of the vagina and uterus, modality of adjuvant radiotherapy, lymphovascular space invasion (LVSI), extent of stromal infiltration, and survival metrics, between two groups: one that received preoperative NACT and one that did not. Data regarding follow-up and patient demographics were systematically obtained from pathology records, inpatient charts, and outpatient medical files. Patients with stage IB2/IIA2 cervical cancer who underwent only staging surgery after administration of NACT were included. Patients who had undergone radical hysterectomy, had a history of non-squamous cell carcinoma of the cervix, or had cancer of any other system in their medical history were excluded. During the course of our research, participants in the NACT group were administered platinum-based combination chemotherapy. The combination of UFT (tegafur/uracil), 5-FU (fluorouracil), and paclitaxel was administered based on the contraindication and comorbidity status of the patients and was applied in 2 or 3 cycles. Patients were assessed following each cycle, and the determination of the number of chemotherapy cycles was made accordingly. In our study, staging surgery included type III radical hysterectomy + bilateral salpingo-oophorectomy + pelvic + paraaortic lymphadenectomy in all patients. Cervical cancer staging was defined according to the 2014 FIGO.

Following the initial therapy, patients underwent a systematic follow-up plan which involved evaluations every three months for the first two years, every six months for the subsequent three years, and then once a year thereafter. During the follow-up examination, the choice of radiological imaging methods was determined based on the patient’s symptoms and physical examination findings, depending on the approaches of the relevant centre. The size of the tumour was determined by measuring its largest dimension. Tumor invasion into the outer half part of the cervical stroma was considered deep stromal invasion. LVSI is defined as tumoral cells or clusters of cells that are attached to a haemotoxylen and eosin-stained vessel wall that encompasses the tumour and the adjacent normal tissue. Uterine invasion was considered as tumour infiltration of the endometrium and/or myometrium above the level of the internal os. Surgical border involvement was positive when the distance between tumor and the distal part of the specimen was ≤ 0.5 cm. Vaginal involvement was defined as tumoral invasion of vagina. The decision to perform bilateral oophorectomy and salpingectomy was considered by the surgeon based on factors such as the patient’s age, menopausal status, and stage of disease. At the time of our study, HPV vaccination was not widespread in our country. As a result, the patient’s HPV and vaccination status are unknown.

Progression-free survival (PFS) was determined from the date of surgical intervention to the emergence of disease relapse or the most recent follow-up occasion. In addition, disease-specific survival (DSS) was calculated from the date of surgery to the date of death attributable to the malignancy or the last date of follow-up.

## Results

**Fig. 1:**
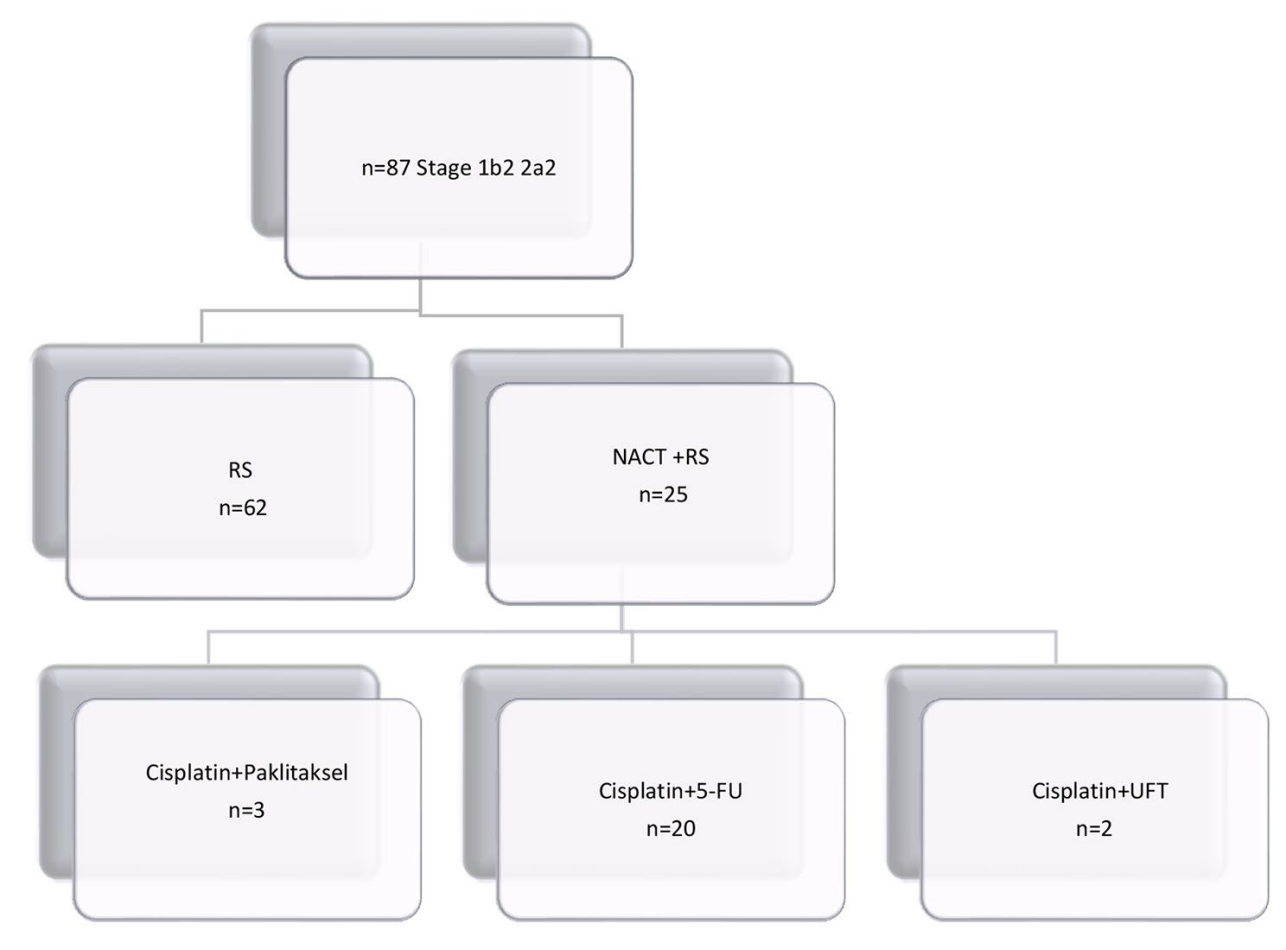
Study flow diagram. RS: Radical surgery NACT: neoadjuvant chemotherapy 5 FU: 5 fluorouracil, UFT: Tegafur and uracil

Totally 87 women with stage IB2 and IIA2 cervical cancer were included in the mentioned period. A total of 25 patients received NACT prior to surgery, while 62 underwent surgery without receiving NACT. Clinical features of entire cohort were presented in Table 1. Out of the patients who received NACT, 80% (20 patients) were given a combination of cisplatin and 5-FU, 12% (3 patients) received both platinum and paclitaxel, and 8% (2 patients) received both cisplatin and UFT (Fig. 1). All patients underwent a type III radical hysterectomy, with the option of bilateral salpingo-oophorectomy, as well as pelvic and/or paraaortic lymphadenectomy. Salpingo-oopherectomy was not performed on 15 patients due to their menopausal status and their personal decision. Postoperatively, none of the patients received chemotherapy.

**Table 1:** Clinical features of entire cohort

Characteristics		Mean ± SD	Median (range)
Age at initial diagnosis		49.6 ± 9.29	48 (31–79)
Tumor size at initial diagnosis (mm)		53.6 ± 9.36	50 (42–80)
Number of removed lymph nodes		52.2 ± 25.52	46 (11–160)
Number of metastatic lymph node		4 ± 6.60	2 (1–31)
		**n**	**%**
FIGO 2014 stage	IB2	78	89.7
	IIA2	9	10.3
Neoadjuvant chemotherapy	Received	25	28.7
	Not received	62	71.3
Parametrial involvement	Negative	63	72.4
	Positive	24	27.6
Surgical border involvement	Negative	82	94.3
	Positive	5	5.7
Vaginal involvement	Negative	68	79.5
	Positive	19	20.5
Lymphovascular space invasion	Negative	35	40.2
	Positive	47	54.0
	Not reported	5	5.7
Depth of stromal invasion	≤ %50	18	20.7
	> %50	68	78.2
	Not reported	1	1.1
Bilateral salpingo-oophorectomy	Not performed	15	17.2
	Performed	72	82.8
Ovarian metastasis ^1^	Negative	70	97.2
	Positive	2	2.8
Uterine involvement	Negative	66	75.9
	Positive	17	19.5
	Not reported	4	4.6
Lymph node metastasis	Negative	63	72.4
	Positive	24	27.6
Site of metastatic lymph node	Only pelvic	21	24.1
	Only paraaortic	-	-
	Pelvic and paraaortic	3	3.5
Adjuvant RT	Not received	30	34.5
	Received	56	64.4
	Not reported	1	1.1
Type of adjuvant RT	CCRT	44	50.6
	Only RT	12	13.8

^1^ Ovarian metastasis was evaluated in 72 patients underwent bilateral salpingo-oophorectomy, SD: Standard Deviation, RT: Radiotherapy, CCRT: Concomitant chemoradiotherapy

Squamous cell carcinoma was present in all patients. A comparative analysis was conducted on clinicopathological parameters between patient groups based on the use or non-use of NACT. (Table 2). There was no difference between the groups in terms of age, tumour size, the number of metastatic lymph nodes, stage, parametrial, surgical border, vaginal, uterine, lymph node involvement, or the type of adjuvant radiotherapy received. The LVSI was more common in patients who did not receive NACT compared to those who had received NACT, with rates of 66.1% and 40%, respectively (*p* = 0.036). Among the patients who did not get NACT 85% had a stromal invasion depth greater than 50%. In contrast, among the patients who did receive NACT, the stromal invasion depth was 56% (*p* = 0.001).

**Table 2:** Comparison of clinico-pathological characteristics between the groups with and without neoadjuvant chemotherapy

Factors		Neoadjuvant Chemotherapy						*p* value
		Not received			Received			
		Mean ± SD	Median	Range	Mean ± SD	Median	Range	
Age		50.0 ± 9.57	47.5	33–79	48.6 ± 8.66	48	31–66	*0.516*
Tumor size at initial diagnosis (mm)		52.1 ± 8.06	50	45–70	52.2 ± 8.33	50	45–70	*0.466*
Total removed lymph node count		52.4 ± 21.7	43.5	24–90	48 ± 21.6	47	15–93	*0.681*
Total metastatic lymph node count		4.2 ± 8.01	1	1–31	3.44 ± 4.42	2	1–15	*0.648*
		**n**	**%**		**n**	**%**		
FIGO 2014 stage	IB2	55	88.7		23	92		*1.000*
	IIA2	7	11.3		2	8		
Parametrial involvement	Negative	48	71.6		19	76		*0.635*
	Positive	19	28.4		6	24		
Surgical border involvement	Negative	63	94		24	96		*1.000*
	Positive	4	6		1	4		
Vaginal involvement	Negative	53	79.1		19	76		*0.779*
	Positive	14	20.9		6	24		
Lymphovascular invasion	Negative	21	33.9		15	60		***0.036***
	Positive	41	66.1		10	40		
Depth of stromal invasion	≤%50	10	15.2		11	44		***0.001***
	>%50	56	84.8		14	56		
Uterine involvement	Negative	49	77.8		22	88		*0.209*
	Positive	14	22.2		3	12		
Lymph node metastasis	Negative	52	77.6		16	64		*0.265*
	Positive	15	22.4		9	36		
Adjuvant RT	Received	40	60.6		18	72		*0.391*
	Not received	26	39.4		7	28		
Type of adjuvant RT	CCRT	30	75		16	88.9		*0.300*
	Only RT	10	25		2	11.1		

SD: Standard Deviation, RT: Radiotherapy, CCRT: Concomitant chemoradiotherapy

The median duration of follow-up was 32 months, with a range of 1 to 228 months. Totally 13 patients (15%) experienced recurrence. Recurrence occurred in 6 patients (9.7%) in the non-NACT group, compared to 7 patients (28%) in the NACT group (*p* = 0.045). Extrapelvic recurrence was observed in 3 patients (12%) in the NACT group, while 3 patients (4.8%) had extrapelvic recurrence in the group who didn’t receive NACT (*p* = 0.348). Among the patients with extrapelvic recurrence, 3 patients had liver metastasis, 2 patients had lung metastasis and 1 patient had bone metastasis.

Totally 6 patients (6.9%) died in the follow-up period. Among these, the deaths of 5 patients (5.7%) were associated with the disease. It has been observed that in the non-NACT group, 2 patients (3.2%) died from the disease, whereas in the NACT group, 3 patients (12%) died from the disease (*p* = 0.140).

Among entire cohort, 2-year PFS and DSS were 82% and 95%, respectively. The factors that may affect PFS are presented in Table 3. In the univariate analysis, only the administration of NACT was found to be associated with PFS (89% vs. %70) (*p* = 0.05). Presence of deep stromal invasion and presence of LVSI differed between the groups who did and didn’t receive NACT (*p* = 0.010 and *p* = 0.036, respectively) and these factors were known to be associated with PFS. Hence, a multivariate analysis was conducted to assess the impact of several factors on PFS, such as the depth of stromal invasion, presence LVSI, and administration of NACT. The results revealed that only NACT emerged as an independent variable associated with poorer PFS. (RR: 5.88; 95% CI: 1.63–21.25; *p* = 0.070) (Table 4).

**Table 3:** Factors predicting DFS, Univariate Analysis

Factors		2-year disease-free survival (%)	*p* Value
Age at initial diagnosis ^1^	≤ 48 years	85	*0.589*
	> 48 years	80	
Tumor size at initial diagnosis ^1^	≤ 50 mm	85	*0.496*
	> 50 mm	79	
2014 FIGO stage	IB2	86	*0.572*
	IIA2	73	
NACT	Not Received	89	*0.05*
	Received	70	
Lymph node metastasis	Negative	84	*0.774*
	Positive	82	
Number of removed lymph node count ^1^	≤ 46	79	*0.350*
	> 46	87	
Lymphovascular space invasion	Negative	90	*0.275*
	Positive	81	
Parametrial involvement	Negative	83	*0.787*
	Positive	84	
Surgical border involvement	Negative	88	*0.714*
	Positive	80	
Vaginal involvement	Negative	87	*0.174*
	Positive	70	
Depth of stromal invasion	≤%50	88	*0.564*
	>%50	85	
Uterine involvement	Negative	83	*0.879*
	Positive	87	
Adjuvant RT	Not received	80	*0.742*
	Received	84	
Type of adjuvant RT	CCRT	85	*0.814*
	Only RT	82	

^1^ Median Value, RT: Radiotherapy, CCRT: Concomitant chemoradiotherapy

**Table 4:** Factors associated with recurrence, multivariate analysis

Risk factor	RR (95%CI)	*p*-value
Neoadjuvant chemotherapy (received vs. not received)	5.88 (1.63–21.25)	***0.007***
Presence of deep stromal invasion (positive vs. negative)	2.55 (0.65-10.00)	0.179
Presence of lymphovascular invasion (positive vs. negative)	2.19 (0.43–11.04)	0.343

## Discussion

The current study compared the oncological outcomes of women diagnosed with stage IB2/IIA2 cervical cancer who underwent radical surgery following neoadjuvant chemotherapy (NACT) versus those who underwent radical surgery alone. Among the prognostic factors examined, only the administration of neoadjuvant NACT exhibited an independent correlation with the recurrence. Patients who had NACT had a risk of recurrence that was six times higher than patients who did not get NACT.

In 2012, Kim et al. reported a meta-analysis of five randomised controlled trials and four observational studies, including patients with surgery after NACT and patients who underwent surgery alone in women diagnosed with stage IBI-IIA cervical cancer. The analysis revealed no statistically significant difference in OS between the study cohorts. However, patients treated with NACT prior to radical surgery had worse OS than those who underwent radical surgery alone (HR: 1.68; 95% CI: 1.12–2.53) [26]. In their retrospective study of 476 patients with stage IB2-IIB disease, Yin et al. found that five-year disease-free survival and OS were significantly better in patients who received NACT followed by radical hysterectomy than in those who received radical hysterectomy alone or concurrent chemoradiotherapy [25]. Another study conducted on women diagnosed with stage IB2-IIB cervical cancer demonstrated that patients who underwent preoperative NACT experienced better DFS and reduced risks of recurrence compared to those who alone underwent radical hysterectomy (NACT group [*p* < 0.001; *p* = 0.013 respectively]) [27]. In 2019, Zhao et al. compared NACT prior to surgery with surgery alone in terms of OS, DFS and locoregional/distant relaps in a review study. In an analysis of 8 studies involving 1544 patients with stage IB2-IIB, the OS rate was increased in the group receiving NACT prior to radical hysterectomy, and locoregional recurrence, parametrial infiltration, and distant metastasis were significantly reduced. However, they noted that further investigation is required in this regard, and stated that the use of NACT prior to radical surgery should be determined by the surgeon according to their experience and clinical judgement [28]. Aforementioned studies included patients with stage IIB disease in their populations and the chemotherapy regimens differed. The inconsistencies between our study and the aforementioned studies may be due to these differences. In contrast, Gong et al. reported a 2-year progression-free survival rate of 93% (95% CI: 0.88–0.98) for NACT and 94.5% (95% CI: 0.91–0.98) for radical hysterectomy alone in patients diagnosed with stage IB2-IIB disease (*p* = 0.659) [29]. Although the study was conducted with a similar number of patients as well as the same stages, the conflicting results between the last two studies may be due to the administration of different chemotherapy regimens.

A Cochrane review, published in 2010 and revised in 2012, demonstrated that both OS and DFS rates are higher in patients treated with NACT prior to surgery in comparison to participants treated with only surgery alone. Population characteristics of the studies included in this review were highly heterogeneous. A review of 6 studies was conducted. Among these trials, two trials involved a population with stage 1B1-1B2, two trials involved a population with stage IB2-IIB, one trial involved a population with stage IB2-IIIB, and one trial involved a population with stage IB2. In these trials, the sample size ranged between 107 and 291 patients. Although cisplatin-based chemotherapy regimens were utilised in all studies, major differences were noted in their methods. The review concluded: “Due to the lack of data and heterogeneity of available studies, NACT should not be recommended before radical surgery except in clinical trials.“ [24].

The clinical outcomes of patients who were diagnosed with cervical carcinoma (stage IB2-IIB) and had surgery following platinum-based NACT were investigated in a multicentre retrospective study, and it has been revealed that pathological response following NACT was found to be an objective prognostic variable for recurrence-free survival and OS. Furthermore, the reports indicated that patients who did not exhibit an optimum response have a 2.757-fold increased risk of recurrence and a 5.413-fold increased risk of mortality [30]. According to the results obtained in this study, prospective studies assessing the pathological response to NACT could be useful for determining the potential benefit of NACT prior to making treatment decisions for early bulky cervical cancer.

Lymph node invasion, positive surgical margin, parametrial infiltration, deep cervical stromal invasion and LVSI were known to be associated with recurrence in cervical cancer. The improved rates of survival could be attributed to the impact of NACT on the risk variables associated with recurrence. Our study demonstrated no significant impact of NACT on parametrial involvement, lymph node metastases, surgical margin involvement, vaginal involvement, and uterine involvement. However, a meta-analysis of 739 patients showed significantly lower rates of nodal involvement (OR, 0.45; 95% CI: 0.29 to 0.70) and parametrial involvement (OR, 0.48; 95% CI: 0.25 to 0.92) in patients who were administered NACT in the preoperative period [31]. Similarly, a study including 142 patients diagnosed with stage IB2-IIB cervical cancer indicated that the NACT + surgery group had lower rates of pelvic lymph node positivity and parametrial involvement [27].

In our study, deep stromal invasion was detected in 56% of cases in NACT group and 84.8% of cases in surgery alone group (*p* = 0.001). LVSI rate was 40% in NACT group and 66.1% in surgery only group (*p* = 0.036). Although NACT decreased the rates of these recurrence risk factors, there was no improvement in radiotherapy requirement rates and survival rates in NACT group. Contrariwise, worse oncological outcomes were observed in patients in NACT group in comparison to patients who were treated with surgery alone. In a randomised controlled trial including patients diagnosed with stage IB cervical cancer LVSI rate was 9.6% in the NACT group and 27.8% in surgery group (*p* = 0.024), and these findings align with the results of our investigation. However, 5-year OS rates were found to be significantly higher in the NACT group compared to radical hysterectomy group (84.6% vs. 75.9%, *p* = 0.011) [32]. The difference in results may be attributed to the retrospective nature of our study and the small sample size of patients in the NACT group.

In our study, 60.6% of cases in the NACT group and 72% of cases who underwent radical hysterectomy only required adjuvant radiotherapy (*p* = 0.391). Similarly, in a study including 288 women diagnosed with stage IB cervical cancer, Eddy et al. found that adjuvant radiotherapy requirement rates were 45% in the NACT group and 52% in surgery alone group (*p* > 0.05) [33]. In contrast, a study including 414 patients demonstrated that preoperative carboplatin-paclitaxel decreased the necessity for adjuvant radiotherapy in women diagnosed with stage IB2-IIA2 cervical cancer (*p* = 0.041) [10]. Similar recurrence rates in the NACT + radical hysterectomy group and only radical hysterectomy group were observed in a phase 3 study conducted by the Gynecological Oncology Group, which included 288 patients (35.7% vs. 33.5%, RR: 0.998). The same study revealed that mortality rates were 36.5% in the NACT group and 35.6% for those who were treated with surgery only (RR: 1.008) [34]. In contrast, we observed 28% recurrence rate in the NACT group and 9.7% recurrence rate for those who were treated with surgery only (*p* = 0.045). During the follow-up period (with a median of 32 months), the overall death rate attributed to the disease was 5.7% in our study. Mortality rate was 12% in the NACT group and 3.2% for those who underwent surgery only (*p* = 0.140).

The optimal modality for NACT is not yet known. A cisplatin-based chemotherapy regimen in combination with ifosphamide, paclitaxel and topotecan may be more efficient in cervical cancer [33]. Matsuma et al. conducted a study including 46 consecutive patients diagnosed with stage IB2-IIB cervical cancer who underwent radical surgery after NACT and followed by adjuvant chemotherapy. The NACT and adjuvant chemotherapy combinations were irinotecan and cisplatin or irinotecan and nedaplatin. The option of radiotherapy treatment was considered in cases of recurrence. The 2-year PFS was 91.2% and the 3-year PFS was 86.1%. Without radiotherapy, 2 patients developed para-aortic lymph node metastases, 2 distant metastases and 3 pelvic recurrences. This treatment has the advantage of reducing radiation-related morbidity in patients diagnosed with stage IB2-IIB cervical cancer and reserving radiotherapy in case of pelvic recurrence [35]. Based on the results of the mentioned studies above, it is crucial to develop treatment methods that do not require the use of radiation for the initial treatment of stages 1B2-IIA2 (FIGO 2014).

The strengths of our study include multicentre design, exclusion of patients with stage IIB disease, and comprehensive evaluation of prognostic factors. However, the retrospective nature of our study, lack of external validation, heterogeneous distribution of patients in the groups, heterogeneous chemotherapy regimens, lack of data on chemotherapy response rates, and failure to calculate overall survival due to the 32-month follow-up period can be considered limitations of our study.

In conclusion, available evidence indicates that NACT should not be routinely administered to patients with early-stage bulky cervical cancer, despite being a well-tolerated treatment modality. Prospective, multi-centred studies with different chemotherapy regimens might clarify the effects of NACT in the management of cervical cancer.

## Data Availability

No datasets were generated or analysed during the current study.

## Declarations

### Informed concent

Patient consent was omitted due to retrospective study design. Researchers analyzed only anonymized data.

## Abbreviations

CCRT: Concomitant chemoradiotherapy
CT: Computerized tomography
DSS: Disease-specific survival
FIGO: International Federation of Obstetrics and Gynecology
HPV: Human papailloma Virus
HR: Hazard Ratio
LVSI: Lymphovascular space invasion
NACT: Neoadjuvant Chemoterapy
MRI: Magnetic Resonance Imaging
PET CT: Positron Emission Tomography
PFS: Progression-free survival
RR: Relative Risk
RT: Radiotherapy
SD: Standard Deviation
OS: Overall Survival
UFT: Tegafur/Uracil
5 FU: 5-Fluorouracil

## References

[1] Arbyn M, Weiderpass E, Bruni L, de Sanjosé S, Saraiya M, Ferlay J (2020). Estimates of incidence and mortality of cervical cancer in 2018: a worldwide analysis. Lancet Global Health.

[2] Liang B-Q, Zhou S-G, Liu J-H, Huang Y-M, Zhu X. Clinicopathologic features and outcome of cervical cancer: implications for treatment. Eur Rev Med Pharmacol Sci. 2021;25(2). 10.26355/eurrev_202101_24631 33577024

[3] Wu SY, Lazar AA, Gubens MA, Blakely CM, Gottschalk AR, Jablons DM (2020). Evaluation of a National Comprehensive Cancer Network guidelines–based decision support Tool in patients with non–small cell Lung Cancer: a Nonrandomized Clinical Trial. JAMA Netw Open.

[4] Sung H, Ferlay J, Siegel RL, Laversanne M, Soerjomataram I, Jemal A (2021). Global cancer statistics 2020: GLOBOCAN estimates of incidence and mortality worldwide for 36 cancers in 185 countries. Cancer J Clin.

[5] Kjær SK, Frederiksen K, Munk C, Iftner T (2010). Long-term absolute risk of cervical intraepithelial neoplasia grade 3 or worse following human papillomavirus infection: role of persistence. J Natl Cancer Inst.

[6] Rodríguez AC, Schiffman M, Herrero R, Hildesheim A, Bratti C, Sherman ME (2010). Longitudinal study of human papillomavirus persistence and cervical intraepithelial neoplasia grade 2/3: critical role of duration of infection. J Natl Cancer Inst.

[7] Cancer ICoESoC (2007). Comparison of risk factors for invasive squamous cell carcinoma and adenocarcinoma of the cervix: collaborative reanalysis of individual data on 8,097 women with squamous cell carcinoma and 1,374 women with adenocarcinoma from 12 epidemiological studies. Int J Cancer.

[8] Dugué P-A, Rebolj M, Garred P, Lynge E (2013). Immunosuppression and risk of cervical cancer. Expert Rev Anticancer Ther.

[9] Oncology FCoG (2014). FIGO staging for carcinoma of the vulva, cervix, and corpus uteri. Int J Gynecol Obstet.

[10] Miriyala R, Mahantshetty U, Maheshwari A, Gupta S. Neoadjuvant chemotherapy followed by surgery in cervical cancer: past, present and future. Int J Gynecologic Cancer. 2022;32(3). 10.1136/ijgc-2021-002531 35256411

[11] Huang Y, Chen L, Cai J, Yang L, Sun S, Zhao J (2022). Neoadjuvant chemotherapy followed by radical surgery reduces radiation therapy in patients with stage IB2 to IIA2 cervical cancer. World J Surg Oncol.

[12] Trattner M, Graf A-H, Lax S, Forstner R, Dandachi N, Haas J (2001). Prognostic factors in surgically treated stage Ib–IIb cervical carcinomas with special emphasis on the importance of tumor volume. Gynecol Oncol.

[13] Park J-Y, Kim D-Y, Kim J-H, Kim Y-M, Kim Y-T, Nam J-H (2011). Outcomes after radical hysterectomy according to tumor size divided by 2-cm interval in patients with early cervical cancer. Ann Oncol.

[14] Sevin BU, Nadji M, Lampe B, Lu Y, Hilsenbeck S, Koechli OR (1995). Prognostic factors of early stage cervical cancer treated by radical hysterectomy. Cancer.

[15] Corrado G, Anchora LP, Bruni S, Sperduti I, Certelli C, Chiofalo B (2023). Patterns of recurrence in FIGO stage IB1-IB2 cervical cancer: comparison between minimally invasive and abdominal radical hysterectomy. Eur J Surg Oncol.

[16] Horn L-C, Fischer U, Raptis G, Bilek K, Hentschel B (2007). Tumor size is of prognostic value in surgically treated FIGO stage II cervical cancer. Gynecol Oncol.

[17] Hebner CM, Laimins LA (2006). Human papillomaviruses: basic mechanisms of pathogenesis and oncogenicity. Rev Med Virol.

[18] Sun H, Xin J, Lu Z, Wang N, Liu N, Guo Q (2013). Potential molecular mechanisms for improved prognosis and outcome with neoadjuvant chemotherapy prior to laparoscopical radical hysterectomy for patients with cervical cancer. Cell Physiol Biochem.

[19] Hwang YY, Moon H, Cho SH, Kim KT, Moon YJ, Kim SR (2001). Ten-year survival of patients with locally advanced, stage IB–IIB cervical cancer after neoadjuvant chemotherapy and radical hysterectomy. Gynecol Oncol.

[20] Panici PB, Scambia G, Baiocchi G, Greggi S, Ragusa G, Gallo A (1991). Neoadjuvant chemotherapy and radical surgery in locally advanced cervical cancer. Prognostic factors for response and survival. Cancer.

[21] Sardi J, Sananes C, Giaroli A, Bayo J, Rueda NG, Vighi S (1993). Results of a prospective randomized trial with neoadjuvant chemotherapy in stage IB, bulky, squamous carcinoma of the cervix. Gynecol Oncol.

[22] Panici PB, Greggi S, Scambia G, Ragusa G, Baiocchi G, Battaglia F (1991). High-dose cisplatin and bleomycin neoadjuvant chemotherapy plus radical surgery in locally advanced cervical carcinoma: a preliminary report. Gynecol Oncol.

[23] Li R, Lu S-t, Si J-g, Liu B, Wang H, Mei Y-y (2013). Prognostic value of responsiveness of neoadjuvant chemotherapy before surgery for patients with stage IB2/IIA2 cervical cancer. Gynecol Oncol.

[24] Rydzewska L, Tierney J, Vale CL, Symonds PR. Neoadjuvant chemotherapy plus surgery versus surgery for cervical cancer. Cochrane Database Syst Reviews. 2012(12). 10.1002/14651858.CD007406.pub3 PMC7175775 23235641

[25] Yin M, Zhao F, Lou G, Zhang H, Sun M, Li C et al. The long-term efficacy of neoadjuvant chemotherapy followed by radical hysterectomy compared with radical surgery alone or concurrent chemoradiotherapy on locally advanced-stage cervical cancer. Int J Gynecologic Cancer. 2011;21(1). 10.1111/IGC.0b013e3181fe8b6e 21330834

[26] Kim H, Sardi J, Katsumata N, Ryu H, Nam J, Chung H (2013). Efficacy of neoadjuvant chemotherapy in patients with FIGO stage IB1 to IIA cervical cancer: an international collaborative meta-analysis. Eur J Surg Oncol (EJSO).

[27] Chen H, Liang C, Zhang L, Huang S, Wu X (2008). Clinical efficacy of modified preoperative neoadjuvant chemotherapy in the treatment of locally advanced (stage IB2 to IIB) cervical cancer: a randomized study. Gynecol Oncol.

[28] Zhao H, He Y, Yang S-L, Zhao Q, Wu Y-M. Neoadjuvant chemotherapy with radical surgery vs radical surgery alone for cervical cancer: a systematic review and meta-analysis. OncoTargets Therapy. 2019:1881–91. 10.2147/OTT.S186451 PMC6413756 30881040

[29] Gong L, Lou J-Y, Wang P, Zhang J-W, Liu H, Peng Z-L (2012). Clinical evaluation of neoadjuvant chemotherapy followed by radical surgery in the management of stage IB2–IIB cervical cancer. Int J Gynecol Obstet.

[30] Gadducci A, Sartori E, Maggino T, Zola P, Cosio S, Zizioli V (2013). Pathological response on surgical samples is an independent prognostic variable for patients with stage Ib2–IIb cervical cancer treated with neoadjuvant chemotherapy and radical hysterectomy: an Italian multicenter retrospective study (CTF study). Gynecol Oncol.

[31] Peng YH, Wang XX, Zhu JS, Gao L (2016). Neo-adjuvant chemotherapy plus surgery versus surgery alone for cervical cancer: Meta‐analysis of randomized controlled trials. J Obstet Gynecol Res.

[32] Cai HB, Chen HZ, Yin HH (2006). Randomized study of preoperative chemotherapy versus primary surgery for stage IB cervical cancer. J Obstet Gynecol Res.

[33] Eddy GL, Bundy BN, Creasman WT, Spirtos NM, Mannel RS, Hannigan E (2007). Treatment of (bulky) stage IB cervical cancer with or without neoadjuvant vincristine and cisplatin prior to radical hysterectomy and pelvic/para-aortic lymphadenectomy: a phase III trial of the gynecologic oncology group. Gynecol Oncol.

[34] Long HJ, Bundy BN, Grendys EC, Benda JA, McMeekin DS, Sorosky J (2005). Randomized phase III trial of cisplatin with or without topotecan in carcinoma of the uterine cervix: a gynecologic Oncology Group Study. J Clin Oncol.

[35] Matsumura M, Takeshima N, Ota T, Omatsu K, Sakamoto K, Kawamata Y (2010). Neoadjuvant chemotherapy followed by radical hysterectomy plus postoperative chemotherapy but no radiotherapy for stage IB2-IIB cervical cancer—irinotecan and platinum chemotherapy. Gynecol Oncol.

